# Indoor PM_2.5_ exposure affects skin aging manifestation in a Chinese population

**DOI:** 10.1038/s41598-017-15295-8

**Published:** 2017-11-10

**Authors:** Anan Ding, Yajun Yang, Zhuohui Zhao, Anke Hüls, Andrea Vierkötter, Ziyu Yuan, Jing Cai, Juan Zhang, Wenshan Gao, Jinxi Li, Manfei Zhang, Mary Matsui, Jean Krutmann, Haidong Kan, Tamara Schikowski, Li Jin, Sijia Wang

**Affiliations:** 1 Ministry of Education Key Laboratory of Contemporary Anthropology, Collaborative Innovation Center for Genetics and Development, School of Life Sciences, Fudan University, Shanghai, 200438 China; 2 Chinese Academy of Sciences Key Laboratory of Computational Biology, Chinese Academy of Sciences–Max Planck Partner Institute for Computational Biology, Shanghai Institutes of Biological Sciences, Shanghai, 200031 China; 3 School of Public Health, Key Lab of Public Health Safety of the Ministry of Education, & Key Lab of Health Technology Assessment of the Ministry of Health, Fudan University, Shanghai, 200031 China; 4 IUF-Leibniz Research Institute of Environmental Medicine, Düsseldorf, 40225 Germany; 5 Fudan-Taizhou Institute of Health Sciences, Taizhou, Jiangsu 225300 China; 6 The Estee Lauder Companies Inc, Melville, NY 10153 United States

## Abstract

Traffic-related air pollution is known to be associated with skin aging manifestations. We previously found that the use of fossil fuels was associated with skin aging, but no direct link between indoor air pollutants and skin aging manifestations has ever been shown. Here we directly measured the indoor PM_2.5_ exposure in 30 households in Taizhou, China. Based on the directly measured PM_2.5_ exposure and questionnaire data of indoor pollution sources, we built a regression model to predict the PM_2.5_ exposure in larger datasets including an initial examination group (N = 874) and a second examination group (N = 1003). We then estimated the association between the PM_2.5_ exposure and skin aging manifestations by linear regression. In the initial examination group, we showed that the indoor PM_2.5_ exposure levels were positively associated with skin aging manifestation, including score of pigment spots on forehead (12.5% more spots per increase of IQR, P-value 0.0371), and wrinkle on upper lip (7.7% more wrinkle on upper lip per increase of IQR, P-value 0.0218). The results were replicated in the second examination group as well as in the pooled dataset. Our study provided evidence that the indoor PM_2.5_ exposure is associated with skin aging manifestation in a Chinese population.

## Introduction

Skin aging is caused by both intrinsic and extrinsic factors. Extrinsic skin aging is known to be affected by various environmental factors including sun exposure, tobacco smoking, and air pollution^1^. For the air pollution exposure, most evidence was established on outdoor air pollutants. Previous studies found significant associations between skin aging and the exposure of NO_2_^2^, PM_10_ and other traffic related particulate matter^3^. On the other hand, few studies have focused on indoor air pollutants, although people generally spend more time indoor than outdoor. An indirect link between indoor air pollution and skin aging was established by a study showing that the use of fossil fuels for cooking was associated with skin aging^4^. However, the direct link between measured indoor air pollutants and skin aging has not been established yet. Recently, the direct measurement of PM_2.5_ concentration by samplers has been widely used in environmental research^5–7^. However, large scale studies with direct measuring of PM_2.5_ exposure is restricted by the substantial costs. Therefore, inferred exposure level based on prediction models which are derived from directly measured data in small scale is a more practical and effective way^8–10^. In this study, using a combination of direct measuring and indirect modeling of the indoor PM_2.5_ exposure level, we estimated the direct association between indoor air pollution and skin aging manifestation.

## Methods and Materials

### Study design and study populations

The participants were recruited out of an on-going large prospective study, the Taizhou Longitudinal Study. The Taizhou cohort study is a cohort of 100,000 adults aged 30–90 years from the general population of Taizhou. Recruitment was through a three-stage stratified random sampling method (see detailed description in Wang *et al*.^9^). This large cohort study aims to investigate the environmental and genetic risk factors for common chronic diseases in China.

In 2012, 1091 participants (aged between 35 and 89 years) out of the large cohort were invited to participate in the questionnaire survey and the skin aging evaluation. See Li *et al*., 2013 for more details on the initial inclusion criteria^4^. Specific to the current study, 217 of the participants were excluded as they have missing data in at least one key factor used to model indoor air pollution exposure (see Assessment of environmental factors). Therefore, 874 of the participants were eventually used as the initial examination group. From the initial examination group, a randomly selected sample of 30 subjects was further selected as a subset for the directly measured year-long residential indoor PM_2.5_ measurements in 3 seasons, autumn/spring, summer and winter. The prediction model was built based on the directly measured subset and was performed on the initial examination group. Then we carried out association analysis between skin aging score and the predicted indoor PM_2.5_.

Using the same method as in the 2012 collection, we further collected skin aging and questionnaire data in additional 1003 participants in 2014. We also predicted their indoor PM_2.5_ exposure and carried out its association with skin aging score. Finally we also combined these 2 dataset as the pooled dataset for further validation.

This study was approved by the ethical committee of Fudan University in Shanghai, China. The Declaration of Helsinki Principles was followed. And all the samples were collected with the informed consent of any participants involved in the study.

### Assessment of environmental factors

A self-administered questionnaire survey was performed to collect information on demographics and potential sources of indoor air pollution in household for the participants. The questionnaire included information of age, gender, education level (primary school/junior high school/senior high school/higher education), body mass index, average daily sun exposure in past decades (in hours), use of solid fuel for cooking (coal, firewood or straw/gas or electricity), smoking (pack-year), passive smoking (yes/no), use of air conditioner (yes/no), average daily time of air conditioner in summer (in hours), the self-reported ventilation level in bedroom and kitchen (good/normal or bad).

The distance between the living place and the main traffic road was calculated by estimation based on the Global Information System (GIS) system. We divided the continuous distance into three levels (short/moderate/long) according to tertiles of the data.

### Assessment of skin aging symptoms

Skin aging symptoms were evaluated by trained personnel according to photo reference scales and on the basis of the skin aging score SCINEXA^TM^ (SCore of INtrinsic and EXtrinsic skin Aging)^11^ in a highly standardized manner as described by Li *et al*.^4^. The number of wrinkles and laxity of eyelids and cheeks were assessed with scores ranging from 0 (not present) to 5 (very severely present). The number of pigment spots was assessed with scores ranging from 0 to 3, with 0 for 0 pigment spots, 1 for 1 to 10 pigment spots, 2 for 11 to 50 pigment spots and 3 for more than 50 pigment spots, respectively. The manifestation of telangiectasia, solar elastosis, cutis rhomboidalis nuchae, Morbus Favre Racouchot, pigment spots on bottom side of the arm and fine wrinkles on the back of hands were evaluated as present or not present.

### Indoor PM_2.5_ sampling and measurements

A total of 30 households were randomly selected taking consideration of geographic coverage. The selected households completed a year-long seasonal PM_2.5_ indoor sampling, including a winter collection in January 2014, a summer collection in August 2014 and an autumn collection in October 2014. During the collection, real-time monitoring devices were set up in the living room at around 1.0–1.2 m high to the ground. The sampling was conducted for one week continuously. Finally, annual indoor PM_2.5_ for each household was calculated by the following equation (unit: µg/m^3^):

1 $${\rm{Annual}}\,{\rm{indoor}}\,{{\rm{PM}}}_{2.5}=({\rm{summer}}\,{\rm{indoor}}\,{{\rm{PM}}}_{2.5}+{\rm{autumn}}\,{\rm{indoor}}\,{{\rm{PM}}}_{2.5}\times 2+{\rm{winter}}\,{\rm{indoor}}\,{{\rm{PM}}}_{2.5})/4$$

Spring and autumn are transition seasons, of which the PM_2.5_ exposure is similar. As a convention, we only measured the PM_2.5_ exposure in one of these two transition seasons.

For PM_2.5_, the particles were collected on Teflon PTEF filters (the pore size 2.0 µm) by pumps (Universal PCXR8, SKC Inc., U.S.A.) connected with the size-selective impactor (personal environmental monitor, cat nr. 761-203B, SKC Inc.,U.S.A.) at the flow rate of 0.002 m^3^/min. Both in the beginning and at the end of each sampling, the pump air flow rates were measured and recorded to ensure a constant level varying within 5%. Also, the filters were weighted before and after the sampling under the constant temperature and relative humidity in the same laboratory after 24 hr of acclimation. In the whole sampling period, environmental temperature and relative humidity were recorded. The PM_2.5_ concentrations in the filter samples were calculated by the formula C_PM2.5_ = (W_2_ − W_1_) × 1000/V_n_, where C_pm2.5_ (µg/m^3^) referred to the concentration of PM_2.5_ in each filter, W_2_ (µg) for the filter weight after sampling, W_1_ (µg) for the filter weight before sampling and V_n_ (m^3^) for the sampling volume transferred in the standard state (0 °C, 101.325 Kpa).

### Regression model to predict indoor PM_2.5_

For the 30 households with directly measured indoor PM_2.5_, we also collected questionnaire information regarding environmental factors potentially affecting the level of indoor PM_2.5_. Using a stepwise regression model, we screened all the factors with the default entry significance level of 0.15 and the exit significance level of 0.15.

Questionnaires including information of the same environmental factors were also collected from the initial examination group and the second examination group. Therefore using the model built based on directly measured indoor PM_2.5_ of the 30 households (randomly selected taking consideration of the geographic coverage), we predicted the level of PM_2.5_ from questionnaire information in the initial examination group and the second examination group.

### Statistical analysis on association between indoor PM_2.5_ and skin aging

By multiple regression analyses, the association between the predicted indoor PM_2.5_ and skin aging effects were estimated in the first, second and in the pooled examination group. Factors associated with skin aging (e.g. age, gender, BMI, pack-years (as a proxy for active smoking), passive smoking, the sun exposure time and the educational level) were included as covariates in the regression analyses.

The adjusted regression coefficients were transformed to arithmetic mean ratios (AMR) for normally distributed skin aging signs with 95% confidence intervals (CI), for log- normally distributed signs to geometric mean ratios (GMR) with 95% CI^3^. Coefficients for the categorical variables were adjusted odds ratios (OR) with 95% confidence intervals (CI).All statistical analyses were performed using SAS 9.3 (SAS Institute Inc., Cary, NC, USA, 2002–2010).

## Results

### Summary statistics of study populations

The characteristics of the initial examination group, the second examination group and the directly measured dataset are presented in Table 1. There were 874 subjects in the initial examination group with 34.4% males (N = 301) and 65.6% females (N = 573), and 1003 subjects in the second examination group with 40.4% males (N = 405) and 59.6% females (N = 598). Subjects in the initial examination group aged between 35 and 89 with an average age of 61.0 years old, while subjects in the second examination group were between 56 and 74 with an average age of 61.8 years old. More than 90% of the subjects had an education level lower than senior high school.

**Table 1:** Description of sample demographics and related environmental factors.

Variables		directly measured dataset (30 subject)	initial examination group (874 subjects)	second examination group (1003 subjects)
**Demographics**				
Age	Mean (SD) Min-max	65.2(7.7) 48–81	61.0(9.6) 35–89	61.8(3.5) 56–74
Male	% Yes (n)	50(15)	34.4(301)	40.4(405)
BMI	Mean (SD)	24.6(4.7)	24.0(3.1)	24.5(3.1)
Education level				
Primary school or lower education	% Yes (n)	83.3(25)	84.0(734)	71.0(712)
Junior high school	% Yes (n)	13.3(4)	12.4(108)	20.0(201)
Senior high school	% Yes (n)	3.3(1)	2.5(22)	6.7(67)
Junior college or higher education	% Yes (n)	0.0(0)	1.1(10)	2.3(23)
Average daily sun exposure in past decades (in h)	Mean (SD)	4.0(2.9)	3.3(2.4)	2.3(1.5)
**Indoor environmental factors**				
Pack-year	Mean (SD)	12.7(20)	6.8(15.5)	7.8(15.0)
Use of air conditioner	% Yes (n)	60(18)	49.8(435)	66.4(666)
Average daily time of air conditioner in summer (in h)	Mean (SD)	2.6(3.4)	2.3(3.4)	2.4(2.7)
Good ventilation condition in bedrooms	% Yes (n)	90.0(27)	91.4(799)	96.6(969)
Good ventilation condition in kitchen	% Yes (n)	80.0(24)	92.0(792)	96.1(962)
Passive smoking	% Yes (n)	66.7(20)	42.2(369)	61.0(612)
Use of solid fuels for cooking	% Yes (n)	36.7(11)	46.3(405)	15.4(154)
Distance to major road				
short (< = 1227 m)	% Yes (n)	40.0(12)	35.7(312)	60.4(606)
Moderate (between 1227 m and 1566 m)	% Yes (n)	36.7(11)	43.0(376)	23.0(231)
Long (>1566 m)	% Yes (n)	23.3(7)	21.3(186)	16.6(166)

All indoor environmental factors are self-reported except the distance to major road which was calculated by estimation based on the Global Information System (GIS) system. And the distance to major road was divided into short, medium and long by the tertiles of the distance to the major roads.

Table 1 also showed the indoor environmental factors which were finally used to predict indoor PM_2.5_. More than 90% of subjects have good ventilation condition in bedrooms. In the initial examination group, the subjects averagely used 2.3 hours air conditioner in one day of summer, 46.3% of them used solid fuel for cooking, and 42.2% of them were exposed to passive smoking. In the second examination group, the subjects stayed in air conditioner environment for 2.4 hours on average, 15.4% of them cooked with solid fuels and 61% of them were exposed to passive smoking. After bidirectional stepwise selection, the prediction model was built based on some of the listed variables, including pack-year, average daily time of air conditioner in summer, good ventilation condition in bedrooms, passive smoking, use of solid fuels for cooking, and distance to major road.

### Measured indoor PM_2.5_ exposure

The PM_2.5_ filter samples were obtained in 30 households for 3 seasons. The annual mean level was 94.3 ± 24.6 µg/m^3^, with 71.4 ± 51.3 µg/m^3^ in the summer, 84.8 ± 26.5 µg/m^3^ in the spring/autumn and 138.5 + 29.5 µg/m^3^ in the winter season, respectively.

**Figure 1:**
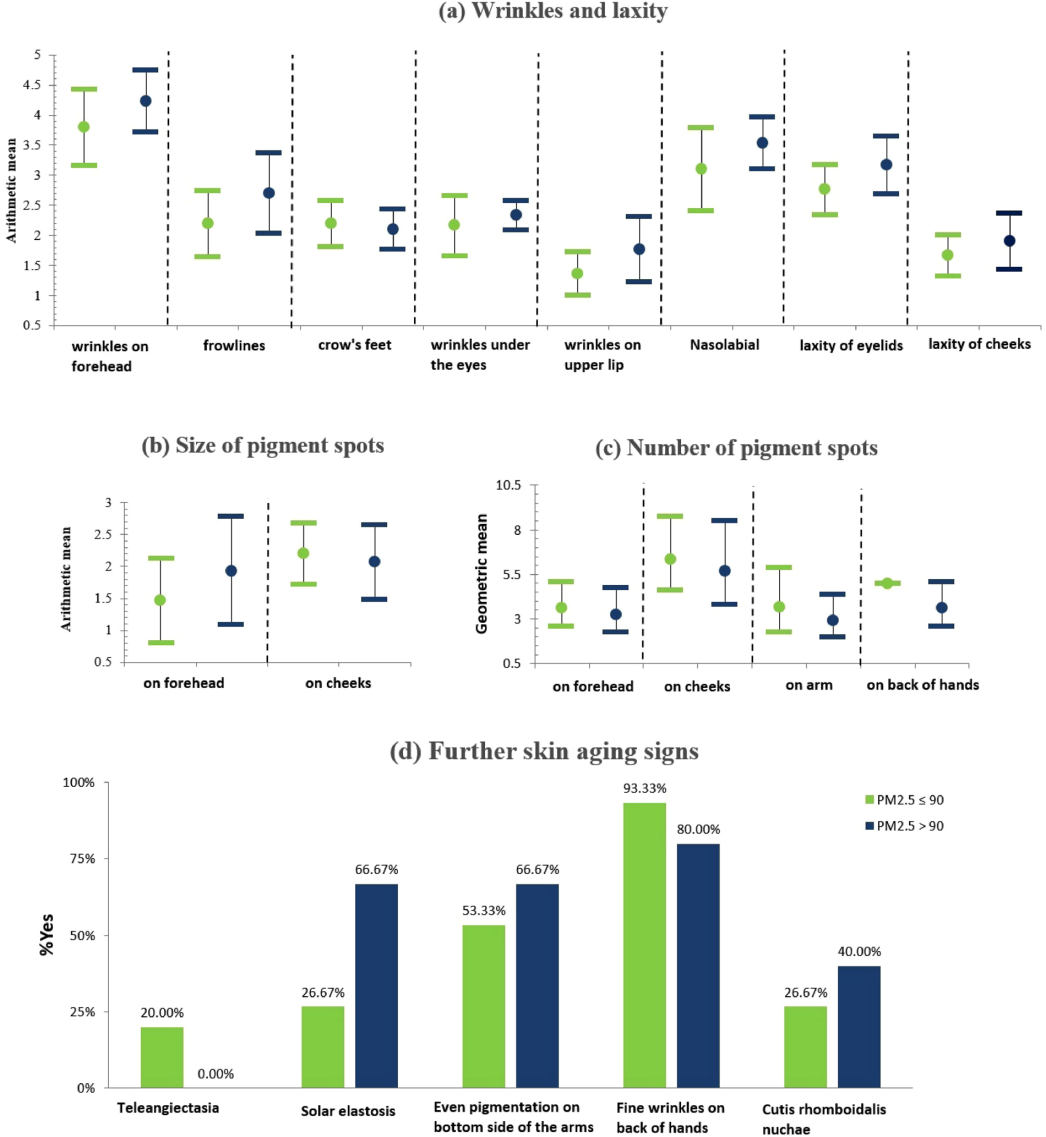
Description of skin aging signs in high indoor PM_2.5_ exposure group and low indoor PM_2.5_ exposure group in the directly measured samples. The study directly measured the indoor PM2.5 of 30 households, and divided them into high PM_2.5_ exposure group (N = 15, annual mean exposure >90 µg/m^3^) and low PM_2.5_ exposure group (N = 15, annual mean exposure ≤90 µg/m^3^). The wrinkles, laxity and size of pigment spots are normally distributed, and therefore arithmetic means (AM) is given; while number of pigment spots is log-normally distributed, and therefore geometric means (GM) is given. 95% CI is presented. Further skin aging manifestations are presented as occurrence respectively.

According to the annual mean level of measured indoor PM_2.5_, we divided the 30 subjects into high PM_2.5_ exposure group (annual mean >90 µg/m^3^, 15 in total) and low PM_2.5_ exposure group (annual mean ≤90 µg/m^3^, 15 in total). The manifestations of skin aging score in these two exposure groups are presented in Fig. 1.

The descriptive comparison of skin aging manifestation in these two exposure groups indicate that the high exposure group have more severe skin aging manifestations than the low exposure group. For example, all wrinkle and laxity traits except crow’s feet are more severe in the high exposure group. Size of pigment spots on forehead and the occurrence of solar elastosis are also more severe in the high exposure group. However, none of the difference reached a statistically significant level, probably because of the small sample size.

### Predicted indoor PM_2.5_ exposure

Stepwise regression analysis including 8 environmental factors (see Table 1) showed that 6 of the factors were significantly associated with a higher PM_2.5_ exposure level. These six factors are number of pack-year, use of air conditioner in summer, ventilation condition in kitchen, passive smoking, use of solid fuels for cooking and distance to major road. The linear prediction model for indoor PM_2.5_ was set up with the R^2^ of 0.657 (Table 2). Ventilation condition in kitchen and use of air conditioner were excluded from the model because of non-significant association. Leave-one-out cross validation was used to validate the linear regression (R^2^ = 0.653, p-value < 0.0001).

**Table 2:** Six environmental factors included in the prediction model.

Variables	Correlation with measured PM_2.5_		Prediction model		
	coefficient	P value	Slope	p-value	R2
Constant			90.65	<0.0001	0.6568
Pack-year	0.47	0.0093	0.42	0.0148	
Passive smoking	0.40	0.0289	24.40	0.0013	
Use of solid fuels for cooking	0.28	0.1285	21.55	0.0100	
Time length of air conditioner in summer	−0.37	0.0423	−2.53	0.0195	
Ventilation condition in bedrooms	−0.26	0.1667	−22.64	0.0379	
Distance to major road	0.07	0.7162	−10.77	0.0347	
Use of air conditioner	−0.29	0.1189	—	—	
Good ventilation condition in kitchen	−0.11	0.5775	—	—	
Outdoor PM_2.5_	0.62	0.0002	—	—	

The prediction model equation with final prediction variables are the following: PM_2.5_ exposure = f(pack-years, passive smoking, use of solid fuels for cooking, time length of air conditioner in summer, ventilation condition in bedrooms, distance to major road).

Using this model, our prediction of indoor PM_2.5_ exposure level ranges from 38.9 µg/m^3^ to 162.8 µg/m^3^ in the initial examination group (mean ± sd = 97.0 ± 20.9), and 31.1 to 149.0 in the second examination group (mean ± sd = 92.5 ± 17.2). These predicted exposure values were then used in the association analysis between indoor PM_2.5_ and skin aging manifestation.

### Association between PM_2.5_ exposure and skin aging manifestation

In the initial examination group, the indoor PM_2.5_ exposure was positively associated with the wrinkles on forehead, wrinkles on upper lip, laxity of eyelids, fine wrinkles on back of hands, and cutis rhomboidalis nuchae. (Tables 3 and S1). Among these 5 skin aging signs, the significant associations between PM_2.5_ exposure and wrinkles on upper lip was replicated in the second examination group. In the pooled dataset, all five significant skin aging signs of the initial examination group were replicated, and pigment spot on forehead also reached significant level (Table 3). The significant association between PM_2.5_ and wrinkles on upper lip can be robustly found in all subsets including the elderlies (>60 years old), males, and females (Table S2).

**Table 3:** The associations between indoor PM_2.5_ exposure and skin aging traits.

		initial examination group (n = 874, per 32.6 µg/m^3^)	second examination group (n = 1003, per 24.4 µg/m^3^)	Pooled dataset (n = 1877, per 28.93 µg/m^3^)
Number and score of pigment spots				
On forehead (score)	AMR(95% CI)	**1.125(1.007,1.243)**	**1.061(1.007,1.116)**	**1.079(1.017,1.141)**
On forehead (number)	GMR(95% CI)	1.07(0.961,1.191)	**1.071(1.002,1.145)**	**1.08(1.01,1.154)**
On cheeks (score)	AMR(95% CI)	1.046(0.961,1.131)	**1.056(1.011,1.1)**	1.047(1,1.095)
On cheeks (number)	GMR(95% CI)	1.013(0.918,1.118)	1.049(0.985,1.117)	1.031(0.969,1.097)
On arm (number)	GMR(95% CI)	0.972(0.869,1.086)	1.072(0.98,1.172)	1.024(0.946,1.109)
On back of hands (number)	GMR(95% CI)	1.052(0.953,1.16)	1.053(0.972,1.141)	1.048(0.976,1.126)
Score of coarse wrinkle				
Wrinkles on forehead	AMR(95% CI)	**1.065(1.021,1.108)**	1.011(0.981,1.041)	**1.033(1.004,1.062)**
Frow lines	AMR(95% CI)	1.02(0.965,1.074)	1.012(0.977,1.047)	1.011(0.976,1.045)
Crow’s feet	AMR(95% CI)	1.006(0.964,1.049)	**1.029(1.003,1.055)**	1.025(0.999,1.052)
Wrinkles under the eyes	AMR(95% CI)	1.001(0.955,1.047)	**1.07(1.04,1.101)**	**1.034(1.004,1.064)**
Wrinkles on upper lip	AMR(95% CI)	**1.077(1.011,1.142)**	**1.087(1.04,1.135)**	**1.094(1.049,1.138)**
Nasolabial	AMR(95% CI)	**1.043(1.015,1.071)**	0.999(0.978,1.021)	1.011(0.991,1.031)
Score of further skin aging symptoms				
Teleangiectasia	OR(95% CI)	0.919(0.629,1.343)	1.099(0.889,1.357)	1.093(0.874,1.367)
Laxity of eyelids	AMR(95% CI)	**1.076(1.036,1.115)**	1.007(0.989,1.024)	**1.032(1.012,1.052)**
Laxity of cheeks	AMR(95% CI)	1.052(0.995,1.108)	0.986(0.964,1.009)	1.008(0.982,1.035)
Presence of further skin aging symptoms				
Solar elastosis	OR(95% CI)	1.099(0.758,1.594)	**1.258(1.005,1.575)**	1.206(0.962,1.512)
Morbus favre racouchot	OR(95% CI)	0.324(0.088,1.194)	0.708(0.238,2.106)	0.549(0.228,1.323)
Even pigmentation on bottom side of the arms	OR(95% CI)	0.962(0.71,1.304)	0.943(0.749,1.188)	0.94(0.765,1.155)
Fine wrinkles on back of hands	OR(95% CI)	1.466(0.963,2.23)	1.176(0.707,1.958)	**1.4(1.017,1.927)**
Cutis rhomboidalis nuchae	OR(95% CI)	**1.636(1.183,2.261)**	1.049(0.828,1.328)	**1.346(1.086,1.669)**

GMR: geometric mean ratio, AMR: arithmetic mean ratio, OR: odds ratio, CI: confidence interval. Data with p-value < 0.05 is marked in bold. A full list of p-values can be found in Table S1.

## Discussion

Our results show evidence that indoor PM_2.5_ exposure might be associated with skin aging manifestation. This is the first study showing a direct link between measured indoor air pollutants and skin aging in Han Chinese. Compared to the well-established environmental factors (e.g. sun exposure and smoking) that play important roles in the development of skin aging signs, indoor air pollution is also a major factor that should not be overlooked.

Although people generally spend more time indoor than outdoor, few studies have focused on indoor air pollutants. One of the main reasons is the high cost of direct measuring of the indoor air pollution exposure levels. In this study, based on the combined use of questionnaire and the directly measured data, we showed an effective way to model PM_2.5_ exposure from a number of environmental factors, including smoking, use of air-conditioner, ventilation, fuel type for cooking and distance to major road. It is worth noting that all the above factors are proven sources of PM_2.5_. It was well established that cigarette smoking is a potent source of fine indoor airborne PM^12^, people close to smokers will be exposed to elevated PM_2.5_ concentrations^6^. Air-conditioning (AC) system was suggested to improve air quality and modify the effects of PM_2.5_^13,14^. Besides, PM_2.5_ was commonly measured to assess exposure to cookstove smoke^15,16^.

In a previous publication, Vierkötter *et al*. showed a direct link between the chronic exposure to traff ic-related particulate matter and the occurrence of prominent skin aging signs especially pigment spots, but also wrinkles in Caucasian women^3^. Li *et al*. then reported an epidemiological evidence that indoor air pollution from cooking with solids fuels, an import source of indoor PM_2.5_, was associated with wrinkles in Chinese women^4^. A recent study has found that exposure to NO_2_ was associated with formation of lentigines in Caucasian and East Asians^2^. Interestingly in this study, the five skin aging traits associated with indoor PM_2.5_ exposure are all wrinkle related features except pigment spots on forehead (wrinkles on forehead, wrinkles on upper lip, laxity of eyelids, fine wrinkles on back of hands, and cutis rhomboidalis nuchae). These results corroborated Li *et al*.’s study that exposure to indoor air pollution from PM_2.5_ is associated with wrinkles, especially for Chinese. Association between outdoor PM exposure and pigment spots, previously reported by Huels *et al*.^2^, was not observed in the initial examination dataset, but only once in the pooled dataset (pigment spot on forehead). Potential reasons could be: (i) the higher baseline risk for pigment spot development in East Asian populations;^4,17^ (ii) genetic differences between Caucasians and Chinese; (iii) particulate matter (PM) might be the leading source of wrinkle formation while gaseous pollutants such as NO_2_ was the major cause of pigment spots formation. Another possible explanation is that indoor PM might differ from outdoor PM due to the difference in constituents and sources.

The major mechanism of ambient PM is the generation of reactive oxygen species (ROS). Particles can serve as carriers for organic chemicals and metals that are capable of localizing in mitochondria and generating ROS directly in mitochondria leading to collagen degradation in human skin and thereby cause wrinkle formation^1,18^. Polycyclic aromatic hydrocarbons (PAHs), adsorbed on the surface of suspended PM, can trigger the arylhydrocarbon receptor (AhR) signaling pathway. The AhR might not only lead to an increased production of ROS, but also indirectly mediate transcriptional expression of genes, which are of known functional relevance for both wrinkle formation and pigment spot formation^19^. These potential mechanisms on how PM could affect skin aging are well supporting our finding that indoor PM could affect wrinkle manifestation in Chinese.

There are several strengths of this study, which need to be discussed, the use of a large cohort from China as well as the direct measurements of indoor air pollution and the use of a validated instrument to measure skin aging, namely the SCINEXA^TM^. There are also some limitations, which need to be considered, indoor air pollution data was only measured in 30 household and then modeled for 1877 households, therefore individual assignment of exposure was fairly coarse could lead to small spatial variation. Secondly, the investigation of the study population was in two steps and it is possible that the initial study population differs to the second examination population.

In conclusion, our results corroborate and extend our previous notion that exposure to indoor air pollutants influences extrinsic skin aging in particular on wrinkle formation.

## Electronic supplementary material

Supplementary Information
