# Supplementary material for: Indoor PM2.5 exposure affects skin aging manifestation in a Chinese population
Source: Sci Rep. 2017 Nov 10;7:15329. doi: 10.1038/s41598-017-15295-8 (PMC5681690; doi:10.1038/s41598-017-15295-8)
Supplement: Supplementary file 1 — Supplementary Information [file 41598_2017_15295_MOESM1_ESM.doc]

**Indoor PM_2.5_ exposure affects skin aging manifestation in a Chinese population**

Anan Ding, Yajun Yang, Zhuohui Zhao, Anke Hüls, Andrea Vierkötter, Ziyu Yuan, Jing Cai, Juan Zhang, Wenshan Gao, Jinxi Li, Manfei Zhang, Mary Matsui, Jean Krutmann, Haidong Kan, Tamara Schikowski, Li Jin, and Sijia Wang

**Table S1. The p-values of associations between indoor PM_2.5_ exposure and skin aging traits.**

|  | Initial examination group  (n=874, per 32.6 µg/m^3^) | Second examination group  (n=1003, per 24.4 µg/m^3^) | Pooled dataset  (n=1877, per 28.93 µg/m^3^) |
| --- | --- | --- | --- |
| Number and score of pigment spots |  |  |  |
| On forehead (score) | **0.0371** | **0.0281** | **0.0127** |
| On forehead (number) | 0.2178 | **0.0433** | **0.0243** |
| On cheeks (score) | 0.2849 | **0.0145** | 0.0521 |
| On cheeks (number) | 0.7948 | 0.134 | 0.3348 |
| On arm (number) | 0.6137 | 0.1298 | 0.555 |
| On back of hands (number) | 0.3142 | 0.2085 | 0.1923 |
| Score of coarse wrinkle |  |  |  |
| Wrinkles on forehead | **0.0037** | 0.4641 | **0.0236** |
| Frow lines | 0.4855 | 0.5081 | 0.5423 |
| Crow's feet | 0.7706 | **0.0308** | 0.0595 |
| Wrinkles under the eyes | 0.9731 | **<.0001** | **0.0245** |
| Wrinkles on upper lip | **0.0218** | **0.0003** | **<.0001** |
| Nasolabial | **0.003** | 0.9477 | 0.2801 |
| Score of further skin aging symptoms | |  |  |
| Teleangiectasia | 0.6629 | 0.3838 | 0.4363 |
| Laxity of eyelids | **0.0002** | 0.4551 | **0.0017** |
| Laxity of cheeks | 0.0727 | 0.246 | 0.5401 |
| Presence of further skin aging symptoms | |  |  |
| Solar elastosis | 0.6181 | **0.0452** | **0.1039** |
| Morbus favre racouchot | 0.0903 | 0.5352 | 0.1818 |
| Even pigmentation on bottom side of the arms | 0.8024 | 0.6199 | 0.5553 |
| Fine wrinkles on back of hands | 0.0742 | 0.5317 | **0.039** |
| Cutis rhomboidalis nuchae | **0.0029** | 0.6936 | **0.0067** |

Data with p-value < 0.05 is marked in bold.

**Table S2. The associations between indoor PM_2.5_ exposure and skin aging traits in subsets of the pooled dataset including the elderlies, males, and females.**

|  |  | pooled dataset | | |
| --- | --- | --- | --- | --- |
|  |  | age>60  (n=1010) | male  (n=706) | female  (n=1171) |
| Number and score of pigment spots |  |  |  |  |
| On forehead (score) | AMR(95% CI) | 1.002(1,1.004) | **1.003(1.001,1.006)** | 1.001(0.999,1.003) |
| On forehead (number) | GMR(95% CI) | 1.002(0.999,1.004) | 1.002(0.999,1.004) | 1.002(0.999,1.004) |
| On cheeks (score) | AMR(95% CI) | 1.001(1,1.003) | **1.002(1,1.004)** | 1(0.999,1.002) |
| On cheeks (number) | GMR(95% CI) | 1.002(1,1.004) | 1(0.997,1.003) | 1.001(0.999,1.003) |
| On arm (number) | GMR(95% CI) | 1.001(0.998,1.004) | **1.005(1.001,1.008)** | **0.997(0.994,1)** |
| On back of hands (number) | GMR(95% CI) | 1.001(0.999,1.004) | 1.002(0.999,1.004) | 1(0.997,1.002) |
| Score of coarse wrinkle |  |  |  |  |
| Wrinkles on forehead | AMR(95% CI) | **1.001(1,1.002)** | **1.001(1,1.002)** | 1.001(1,1.002) |
| Frow lines | AMR(95% CI) | 1(0.999,1.002) | 1(0.999,1.002) | 1(0.999,1.001) |
| Crow's feet | AMR(95% CI) | 1.001(1,1.002) | 1(0.999,1.001) | 1(0.999,1.001) |
| Wrinkles under the eyes | AMR(95% CI) | **1.001(1,1.002)** | 1.001(0.999,1.002) | 1.001(1,1.002) |
| Wrinkles on upper lip | AMR(95% CI) | **1.003(1.001,1.004)** | **1.002(1.001,1.004)** | **1.002(1.001,1.003)** |
| Nasolabial | AMR(95% CI) | 1(1,1.001) | 1(0.999,1) | 1(0.999,1.001) |
| Score of further skin aging symptoms | |  |  |  |
| Teleangiectasia | OR(95% CI) | 1.002(0.995,1.01) | 0.998(0.99,1.007) | 1.002(0.994,1.009) |
| Laxity of eyelids | AMR(95% CI) | 1.001(1,1.001) | 1(0.999,1.001) | **1.001(1,1.001)** |
| Laxity of cheeks | AMR(95% CI) | 1(0.999,1.001) | 0.999(0.998,1) | 1(0.999,1.001) |
| Presence of further skin aging symptoms | |  |  |  |
| Solar elastosis | OR(95% CI) | 1.005(0.998,1.012) | 1.004(0.994,1.013) | 1.003(0.996,1.01) |
| Morbus favre racouchot | OR(95% CI) | 1.001(0.973,1.029) | 1.013(0.986,1.04) | **0.947(0.902,0.994)** |
| Even pigmentation on bottom side of the arms | OR(95% CI) | 0.998(0.991,1.005) | 0.998(0.99,1.007) | 0.994(0.988,1.001) |
| Fine wrinkles on back of hands | OR(95% CI) | 1.003(0.986,1.02) | 1.007(0.993,1.021) | **1.012(1.001,1.024)** |
| Cutis rhomboidalis nuchae | OR(95% CI) | 1.007(1,1.014) | **1.01(1.001,1.019)** | 1.004(0.998,1.011) |

Data with p-value < 0.05 is marked in bold.
